# Benthic-pelagic coupling in the Barents Sea: an integrated data-model framework

**DOI:** 10.1098/rsta.2019.0359

**Published:** 2020-08-31

**Authors:** Felipe S. Freitas, Katharine R. Hendry, Sian F. Henley, Johan C. Faust, Allyson C. Tessin, Mark A. Stevenson, Geoffrey D. Abbott, Christian März, Sandra Arndt

**Affiliations:** 1 School of Earth Sciences, University of Bristol, Wills Memorial Building, Queen's Road, Bristol BS8 1RJ, UK; 2 BGeosys, Department of Earth and Environmental Sciences, CP 160/02, Université Libre de Bruxelles, 1050 Brussels, Belgium; 3 School of GeoSciences, University of Edinburgh, James Hutton Road, Edinburgh EH9 3FE, UK; 4 Schoof of Earth and Environment, University of Leeds, LS2 9TJ Leeds, UK; 5 Department of Geology, Kent State University, Kent, OH, 4424, USA; 6 School of Natural and Environmental Sciences, Newcastle University, Drummond Building, Newcastle upon Tyne NE1 7RU, UK

**Keywords:** organic matter reactivity, degradation rates, nutrient fluxes, reaction-transport model, seafloor, continental shelf

## Abstract

The Barents Sea is experiencing long-term climate-driven changes, e.g. modification in oceanographic conditions and extensive sea ice loss, which can lead to large, yet unquantified disruptions to ecosystem functioning. This key region hosts a large fraction of Arctic primary productivity. However, processes governing benthic and pelagic coupling are not mechanistically understood, limiting our ability to predict the impacts of future perturbations. We combine field observations with a reaction-transport model approach to quantify organic matter (OM) processing and disentangle its drivers. Sedimentary OM reactivity patterns show no gradients relative to sea ice extent, being mostly driven by seafloor spatial heterogeneity. Burial of high reactivity, marine-derived OM is evident at sites influenced by Atlantic Water (AW), whereas low reactivity material is linked to terrestrial inputs on the central shelf. Degradation rates are mainly driven by aerobic respiration (40–75%), being greater at sites where highly reactive material is buried. Similarly, ammonium and phosphate fluxes are greater at those sites. The present-day AW-dominated shelf might represent the future scenario for the entire Barents Sea. Our results represent a baseline systematic understanding of seafloor geochemistry, allowing us to anticipate changes that could be imposed on the pan-Arctic in the future if climate-driven perturbations persist.

This article is part of the theme issue ‘The changing Arctic Ocean: consequences for biological communities, biogeochemical processes and ecosystem functioning’.

## 1. Introduction

**Figure 1.**
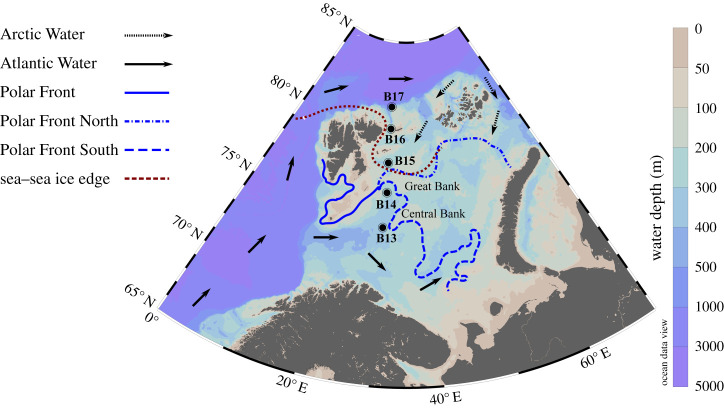
The Barents Sea and location of processed stations along the 30° E S–N transect during the JR16006 cruise, July–August 2017. Water masses and Polar Front positions adapted from Oziel *et al*. [4,5]. Sea–sea ice edge position adapted from Norwegian Meteorological Institute ice charts (mid July 2017; https://cryo.met.no/archive/ice-service/icecharts/quicklooks/2017/). Map produced using Ocean Data View [6]. (Online version in colour.)

Continental shelves play a significant role in organic matter (OM) and nutrient recycling [1,2], and high productivity arctic shelves are important hotspots for benthic processing [3]. The Barents Sea (figure 1) covers nearly one-third of the Arctic shelves and accounts for about half of Arctic primary productivity (PP) [7]. Many factors contribute to the high productivity in the Barents Sea, estimated as 93 g C m^−2 ^yr^−1^ [8]. Complex oceanographic dynamics have a major influence on the Barents Sea PP. This is mostly driven by the inflow of nutrient-rich, relatively warm Atlantic Water (AW) and its modification over the shelf through atmospheric heat exchange. Furthermore, AW interacts with cold Arctic Water (ArW) giving rise to the Polar Front (PF) along the Central and Great banks. Additionally, sea ice dynamics, marked by strong seasonal and interannual variability, also play an important role in shaping OM productivity patterns [9–14]. Such factors lead to an overall elevated PP in the AW-dominated southern shelf (120 g C m^−2^ yr^−1^), which is two-fold higher than PP of the northern shelf [7,8]. Spatio-temporal PP patterns dictate OM vertical export and the quality of OM delivered to the seafloor [4,15,16], which is reflected in benthic ecosystem structure [17,18]. Therefore, climate change-driven perturbations to this Arctic system can lead to large, yet unquantified, disruptions of ecosystem functioning in the Barents Sea. In fact, there is evidence of long-term summer sea ice retreat [12,19], resulting in sea ice loss and an expansion of the seasonal ice zone [10]. Recent modification of summer sea ice dynamics has been attributed to strengthening of AW inflow in the southern Barents Sea region, forcing the PF towards the northern and north-eastern shelf, resulting in an ‘Atlantification’ of the northern Barents Sea [5,11,13]. In addition to sea ice retreat, disruption to water column stratification and associated deepening of the mixed layer result in increased PP due to enhanced nutrient recycling and replenishment, as well as longer duration of blooms [8]. Thus, PP patterns are likely to change significantly, modifying OM export and benthic ecosystem structure [7]. If perturbations to Barents Sea oceanographic conditions persist, the present-day southern shelf conditions might represent the future scenario of the northern shelf, and possibly the wider Arctic Ocean [10,19,20]. In recent years (e.g. 2018), ice-free conditions have been established over the entire Barents Sea shelf during summer months (Norwegian Meteorological Institute, https://cryo.met.no/archive/ice-service/icecharts/quicklooks/2018/). This is critical, as predictions anticipate ice-free summers every year for the Barents Sea by 2050 if present trends continue [12].

The long-term changes occurring in the Barents Sea bring with them huge uncertainty for OM and nutrient cycling. Present-day OM recycling and burial are mostly dictated by the timing of blooms, as well as match or mismatch of grazing in the water column, including processes controlling the quality and quantity of OM settling on the sediment [7,16]. What will happen to seafloor processes upon such changes in bloom dynamics and OM characteristics is still an open question. Understanding how changes in PP will systematically impact benthic-pelagic coupling is crucial in constraining the future state of the Arctic Ocean. Despite such motivation, we still lack a mechanistic understanding of the processes governing OM and nutrient processing at the present-day Barents Sea seafloor. This is a vital piece of the puzzle that the future Arctic Ocean represents. Here, we address this gap by developing a coupled data-model base-line study, which builds on a comprehensive geochemical dataset obtained across a water mass and sea ice gradient across the Barents Sea shelf during the summer of 2017 (figure 1). We aim to understand what controls OM burial and nutrient recycling and how they might impact biological production in the wider Arctic Ocean. Our specific objectives are to quantify OM reactivity patterns, and to estimate degradation rates and nutrient fluxes. We develop an integrated framework to interrogate the drivers of those processes and the controls of benthic-pelagic coupling in the Barents Sea. This allows us to establish grounds for future studies investigating how environmental changes, such as those mediated by climate change, can modify Arctic Ocean biogeochemistry.

## 2. Methods

We investigate five locations on the Barents Sea shelf along a 30° E transect (74° N – 81° N) to reconstruct and quantify sedimentary OM dynamics (table 1 and figure 1), combining observational data and modelling approaches.

**Table 1.** Geographical positions and bottom water physical and chemical characteristics [21,22] of sites along the 30° E S–N transect visited in July–August 2017.

				bottom water (approx. 10 m above seafloor)		
site	latitude °N	longitude °E	depth m	temperature °C	salinity	dissolved O_2_ µM
B13	74.4666	30.0003	355	1.76	35.014	318.7
B14	76.4994	30.287	290	1.94	35.010	300.8
B15	78.2143	30.0007	330	−1.50	34.900	338.7
B16	80.1521	29.916	294	−1.45	34.682	343.7
B17	81.4018	29.5066	291	1.75	34.901	317.4

### (a) Data acquisition

Sampling took place in July–August 2017 on-board RRS *James Clark Ross* during cruise JR16006, at seafloor sites with similar sediment types (mainly silty mud) and water depths (280–370 m). The transect also crossed the PF and the average winter and summer sea ice edge [21]. Vertical profiles of physical and chemical properties of the water column were measured using sampling rosette equipped with a SeaBird SBE911Plus CTD package and 24 × 20 l Niskin bottles for discrete sampling over the water column depth [21,22]. Intact sediment cores were sampled using a Megacorer (BODC, Southampton, UK) and processed on board within at most an hour after retrieval. Porewaters were extracted directly from the cores in regular depth intervals (1 cm to 2.5 cmbsf, 2 cm to 20.5 cmbsf, 5 cm to base) using Rhizons (Rhizosphere Research Products, NL; 0.15 µm pore size) with vacuum applied by plastic syringes with stoppers. Parallel sediment cores were sliced at regular depth intervals (0.5 cm to 2 cmbsf; 1 cm to base). Porewater and sediment sampling were performed in triplicate (i.e. three independent multicore deployments) and subsamples were preserved on board according to each analytical requirement [21]. Porewater nutrients concentrations ${\text{( NO}}_{3}^{-},{\text{NH}}_{4}^{+},{\text{ PO}}_{4}^{3-})$ were analysed on-board using a Lachant Quikchem 8500 flow injector analyser standardized using international certified reference materials for nutrient seawater (KANSO Ltd., Japan) [21]. Sedimentary bulk organic carbon contents were analysed onshore. Dry bulk sediment samples were acidified (4 M HCl) and analysed on a Leco CS230 elemental analyser at Newcastle University [23]. Solid-phase Mn and Fe contents were determined by wavelength dispersive X-ray fluorescence (XRF) using a Philips PW-2400 WD-XRF spectrometer calibrated with 53 geostandards at the University of Oldenburg. Analytical precision and accuracy were better than 5% as checked by in-house and international standards [24]. Porewater Mn and Fe concentrations were analysed using a Thermo Scientific iCAP 7400 Radial ICP-OES at the University of Leeds. Instrument uncertainty was less than 3%. For model inputs, porewater Fe and Mn ICP results were assumed to represent dissolved Fe^2+^ and Mn^2+^. Sediment porosity was determined gravimetrically by determining the water content of sediment samples and assuming a particle density of 2.65 g cm^−3^. We use these data (table 2) to inform a state-of-the-art reaction-transport model (RTM), which allows us to constrain OM dynamics at the Barents Sea seafloor.

**Table 2.** Site-specific upper boundary conditions prescribed to the steady-state RTM developed for the Barents Sea 30° E S–N transect.

	TOC	O_2_	${\text{NO}}_{3}^{-}$	Mn_(s)_	Fe_(s)_	${\text{SO}}_{4}^{2-}$	${\text{NH}}_{4}^{+}$	${\text{PO}}_{4}^{3-}$	Mn^2+^	Fe^2+^
site	wt%	µM	µM	wt%	wt%	mM	µM	µM	µM	µM
B13	2.21	100	12	0.04	0.83	28	0	0	0	0
B14	2.50	50	12	0.11	1.82	28	0	0	0	0
B15	1.80	75	12	0.48	1.70	28	0	0	0	0
B16	1.58	200	12	0.63	1.52	28	0	0	0	0
B17	1.70	125	12	0.62	1.38	28	0	0	0	0

### (b) Model description

**Figure 2.**
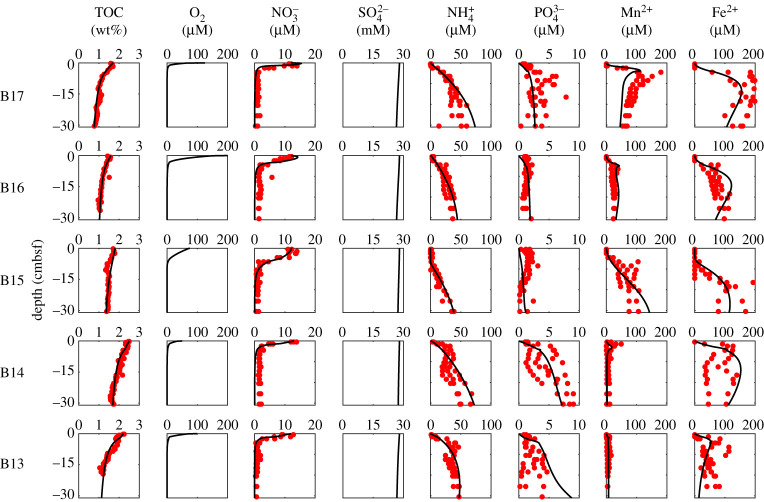
Site-specific (rows: B13–B17) data-model best-fits assuming steady-state depositional conditions for concentration depth profiles (columns: total organic carbon, oxygen, nitrate, sulfate, ammonium, phosphate, dissolved manganese and dissolved iron). Circles denote measured data for July–August 2017 dataset (JR16006 cruise) and solid lines represent RTM outputs based on organic matter reactivity parameters (*a* and *v*). Oxygen and sulfate measurements not available for this dataset. See electronic supplementary material, figure S1 for oxygen concentration profiles determined in July 2019 for comparison. (Online version in colour.)

We employ the Biogeochemical Reaction Network Simulator (BRNS) [25,26] to first reconstruct the sedimentary OM dynamics, and then to quantify benthic processes. The BRNS is an adaptive simulation environment, suitable for large, mixed kinetic-equilibrium reaction networks, which has been successfully adopted on different spatial and temporal scales [27–30]. The model is based on the vertically resolved mass conservation equation for solids and dissolved species in porous media (equation (2.1)) [31,32]:

2.1 $$\frac{\partial \sigma C_{i}}{\partial t}=\frac{\partial }{\partial z}\left(D_{bio}\sigma \frac{\partial C_{i}}{\partial z}+D_{i}\sigma \frac{\partial C_{i}}{\partial z}\right)-\frac{\partial \sigma \omega C_{i}}{\partial z}+a_{i}\sigma (C_{i}(0)-C_{i})+\underset{j}{\sum }s_{i}^{j}R^{j},$$

where *C_i_* is the concentration of the species *i*, *t* denotes time, *z* is the sediment depth. For solid species, the porosity term is given by σ=(1−φ), whereas for dissolved species porosity assumes σ=φ. The effective molecular diffusion coefficient of dissolved species is given by *D_i_* (*D_i_* = 0 for solid species). Here, we assume typical molecular diffusion coefficients [33]. *D*_bio_ represents the bioturbation diffusion coefficient. *D*_bio_ values were derived experimentally in parallel to our sampling [34]. The bioirrigation coefficient is denoted by *a_i_* (*a_i_* = 0 for solid species). Since we do not have a quantitative constraint on bioirrigation for this region, we assume global values [28]. Sedimentation rate is given by *ω*. Accurately reproducing the timescale within the model domain is challenging, since the sedimentation rates are poorly constrained in the Barents Sea. Estimates range from 0.02 to 0.07 mm yr^−1^ based on mollusc shells radiocarbon measurements for sediment depth greater than 25 cmbsf [35–37] to 0.3–1.7 mm yr^−1^ based on ^210^Pb and ^137^Cs for shallow sediment depths < 15 cmbsf [38,39]. Here, we assume the nearest site-specific ^210^Pb estimates (0.5–0.6 mm yr^−1^ [38]; see electronic supplementary material, table S4), since those values reflect recent sedimentation in the uppermost sediment layers and allow the RTM to best reproduce the observations. Since such estimates are linear accumulation rates, they do not account for the downcore variability and spatial heterogeneity (e.g. TOC fluctuations in B15) we see in our dataset (figure 2). Additionally, sediment mixing in the uppermost layers modifies the timescale of sediment burial [40]. We adopt mixed-depth layer estimates of between 2 and 5 cm (see electronic supplementary material, table S4) based on macrofaunal experiments carried out in parallel to our sampling [34]. Such values agree with radionuclides (^210^Pb and ^234^Th) estimates which reflect short-lived biological mixing [39,40].

The sum of consumption/production rates *j* is given by $\sum_{j}s_{i}^{j}R^{j}$, where the stoichiometric coefficient of species *i* is given by $s_{i}^{j}$ for the kinetically controlled reaction *j*, with rate *R^j^*. The model developed here assumes steady-state depositional conditions, thus it represents long-term trends, and therefore it does not explicitly capture seasonal features. The BRNS accounts for fluxes and transformations of OM, the full suite of terminal electron acceptors and the most relevant reduced species. Briefly, the model accounts for primary redox reactions (aerobic respiration, denitrification, manganese reduction, iron reduction, sulfate reduction and methanogenesis), secondary redox reactions (nitrification, Mn^2+^, Fe^2+^ and sulfides oxidation), as well as mineral precipitation and dissolution (FeS and FeS_2_) and equilibrium (${\text{NH}}_{4}^{+}$ and ${\text{PO}}_{4}^{3+}$ sorption and desorption, the equilibria of the carbonate-, the total sulfide- and total borate-systems) reactions. For a detailed reaction network, see electronic supplementary material, tables S1 and S2.

The rates of OM degradation *R*_Corg_ are calculated assuming a reactive continuum of OM compounds and first-order kinetics with respect to the electron donor. It is assumed that bulk OM is continuously distributed over a range of reactivity *k*. Due to the fast depletion of most reactive compounds, *k* decreases during degradation, and thus reflects the widely observed reactivity decrease with burial time/depth/age, termed reactive continuum model (RCM) [32,41]. Due to limitations of the RCM approach in calculating sediment age within the sediment mixed layer [42,43], the continuum OM distribution is approximated by discretizing the continuous distribution of OM over the reactivity spectrum using a multi-G approach [44] that accounts for 200 fractions within the bioturbated zone. In the RCM, the bulk OM reactivity *k* distribution and downcore evolution are determined by the free, positive parameters *a* and *v* (equation (2.2)):

2.2 $$k(z)=\frac{v}{a+\text{age}(z)}.$$

The *v* parameter is a dimensionless, scaling parameter of bulk OM distribution, whereas *a* is a free shaping parameter, which is generally interpreted as an average lifetime of OM and expressed in years [32]. In general, high *v* and low *a* values represent a more reactive, yet more heterogeneous OM mixture. Such a combination yields high *k* at the sediment-water interface (SWI) but results in a fast decrease in *k* with burial depth/time as a result of a rapid loss of the most reactive components of bulk OM. By contrast, low *v* and high *a* values result in low *k* at the SWI and a slow decrease in *k* with burial [45].

We constrain *a* and *v* based on the best-fit between the BRNS simulations and the concentration depth profiles measured during the summer of 2017. Since RTM simulations assume steady-state conditions, they capture and reproduce long-term trends. It is unclear how the strong seasonal dynamics in primary production [7,9,12,15,18] will affect benthic processes. Several integrative, time-series studies on the Antarctic shelf show that intense seasonality in primary production is heavily dampened in sediments on the Western Antarctic Peninsula [46]. This decoupling may result in part from the accumulation of a persistent sediment ‘food bank’ that buffers the benthic ecosystem from the seasonal variability of the water column [47].

Oxygen concentrations at the SWI play a major role in OM and nutrient cycling at the seafloor [3,48], and are commonly incorporated into RTMs [26,28,33]. Our RTM explicitly accounts for OM aerobic respiration, as well as oxygen-mediated re-oxidation of ${\text{NH}}_{4}^{+}$, $\text{M}{\text{n}}^{2+}$, $\text{F}{\text{e}}^{2+}$, $\text{H}{\text{S}}^{-}$, ${\text{H}}_{\text{2}}\text{S}$ and FeS (see electronic supplementary material, tables S1 and S2). Since SWI and downcore oxygen measurements are not available for our dataset, we initially assumed bottom water oxygen values (O_2_ > 300 µM [22]; table 1). Nevertheless, such values were shown to be unsuitable upper boundary conditions for oxygen when considering their impacts on the depth-evolutions of ${\text{NO}}_{3}^{-}$, ${\text{NH}}_{\text{4}}^{\text{ + }}$, $\text{M}{\text{n}}^{2+}$ and $\text{F}{\text{e}}^{2+}$, which suggests that SWI oxygen levels are lower than bottom water values [49]. Therefore, we prescribed lower than bottom water oxygen levels (table 2) to enable the RTM to better reproduce dissolved species depth profiles (figure 2). Such an approach may result in uncertainty when constraining fluxes across the SWI. Nevertheless, oxygen depth profiles measured in the same locations in the summer of 2019 (see electronic supplementary material figure S1) show a clear drop in concentrations between bottom water and SWI, as well as shallow penetration depths [50] which confirms that our initial approach of inversely constraining oxygen penetration depths on the basis of ${\text{NO}}_{3}^{-}$, ${\text{NH}}_{\text{4}}^{\text{ + }}$, $\text{M}{\text{n}}^{2+}$ and $\text{F}{\text{e}}^{2+}$ is valid. Additionally, it has been suggested that macrofauna respiration in sediments [48] represents a major component of sediment oxygen consumption on the Barents Sea seafloor [17,51]. Hence, our assumption of lower than bottom waters SWI oxygen concentrations is plausible.

Model parameterization often relies on global scale compilation of sometimes poorly constrained reaction rate constants. We initially assumed typical global values for secondary redox reaction biomolecular rate constants [26,28,33]. However, our initial tests showed that such values are unsuitable for reproducing our measured downcore concentration profiles (figure 2). Therefore, site-specific biomolecular rate constants for ammonium oxidation, as well as those for manganese, iron and phosphorus cycling were prescribed based on RTM outputs and our measured porewater depth profiles (see electronic supplementary material, table S5). This suggests that local/regional microbial processes not explicitly described in our RTM have a substantial influence on OM and nutrient recycling.

Based on RTM best-fit, we calculate the rates of OM degradation *R*_Corg_, integrated over the entire model domain $\sum R_{\text{Corg}}$ (equation (2.3)), as well as the relative contribution of each respiration pathway to the total rates of OM respiration [28]

2.3 $$\sum R_{\text{Corg}}=\underset{n}{\sum }\int_{0}^{L}r_{n}\text{d}x,$$

where *L* is the length of the model domain, *n* denotes the respiration pathway and *r_n_* represents the reaction rate of each pathway. Additionally, we derive from the data-model best-fit the nutrient fluxes *J_i_* (equation (2.4)) across the SWI

2.4 $$J_{i}=J_{i,\text{Advection}}+J_{i,\text{Diffusion}}+J_{i,\text{Bioturbation}}+J_{i,\text{Bioirrigation}}.$$

## 3. Results and discussion

Best-fit simulation results ${\text{(TOC, NO}}_{\text{3}}^{-}{\text{, NH}}_{\text{4}}^{\text{ + }}{\text{, PO}}_{\text{4}}^{\text{3}-}\text{, M}{\text{n}}^{2+}\text{, F}{\text{e}}^{2+})$ for the five investigated stations along the S–N transect exhibit good agreement with observational data (figure 2). Our RTM results derived from best fits (table 3) reveal a series of insights into OM cycling in the Barents Sea. We explore OM reactivity patterns and their links with the Barents Sea oceanographic conditions. Additionally, we discuss OM degradation rates and SWI nutrient fluxes and explore how these processes are influenced by reactivity patterns.

**Table 3.** Model-derived organic matter (OM) degradation dynamics along the Barents Sea 30° E S-N transect derived from July–August 2017 dataset: OM reactivity shaping parameter, *a*; OM reactivity scaling parameter, *b*; OM reactivity at the sediment-water interface (equation (2.2)), *k*_SWI_; total heterotrophic OM degradation rates integrated over the uppermost 100 cm of sediment column, i.e. depth-integrated rates, $\sum R_{\text{Corg}}$; ammonium benthic fluxes, $J_{\text{N}{\text{H}}_{\text{4}}}$; phosphate benthic fluxes, $J_{\text{P}{\text{O}}_{\text{4}}}$.

	OM reactivity parameters			benthic-pelagic coupling		
	*a*	*b*	*k*_SWI_	$\sum R_{\mathrm{Corg}}$	$J_{{\mathrm{NH}}_{4}}$	$J_{{\mathrm{PO}}_{4}}$
site	yr	–	yr^−1^	µmol C cm^−2^ yr^−1^	µmol ${\text{NH}}_{4}^{+}$ cm^−2 ^yr^−1^	µmol ${\text{PO}}_{4}^{3-}$ cm^−2^ yr^−1^
B13	20	0.150	7.5 × 10^−3^	108.3	1.47	0.016
B14	20	0.090	4.5 × 10^−3^	90.5	8.85	0.045
B15	100	0.100	1.0 × 10^−3^	31.6	0.05	0.001
B16	10	0.090	9.0 × 10^−3^	86.0	1.39	0.008
B17	20	0.200	2.0 × 10^−2^	122.5	2.69	0.012

### (a) Spatial patterns of apparent organic matter reactivity along the S–N transect

**Figure 3.**
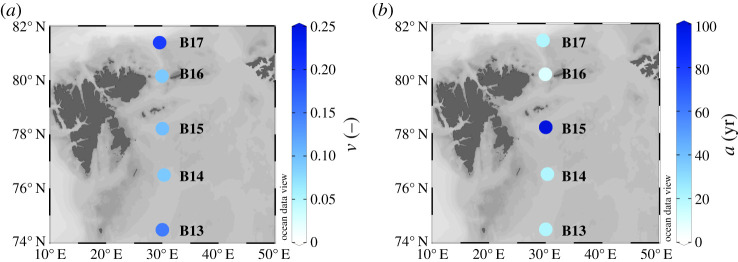
Distributions of organic matter reactivity parameters along the 30° E S–N transect. (*a*) Scaling parameter *b*, (*b*) shaping parameter *a* (yr). (Online version in colour.)

Our inverse modelling approach allows us to obtain bulk OM reactivity parameters (equation (2.2)), and thus explore the environmental controls on apparent OM reactivity (i.e. parameters *a* and *v*). The scaling parameter *v* exhibits a narrow range of values, *v* = 0.090–0.200 (table 3 and figure 3*a*). This interval falls within the global range found across many depositional environments and temporal scales [45]. The *v*-values for Barents Sea sediments thus exert a minor influence on the spatial heterogeneity of apparent bulk OM reactivity and its sediment depth profile. The central portion of the Barents Sea (B14 – B16) displays the lowest *v*-values (*v* = 0.090–0.100), whereas B13 and B17 exhibit the highest *v* (*v* = 0.150–0.200), suggesting a slightly higher apparent reactivity of bulk OM in those areas. Interestingly, B13 and B17 are both areas influenced by AW [5,9,16,18,52]. This influence is evident from bottom water temperature and salinity observations at B17 [22], which show an intrusion of warmer and more saline AW along the northern Barents Sea shelf [5]. By contrast, the central Barents Sea shelf, the vicinity of the PF and the summer sea ice edge are characterized by lower *v*, and thus reveal a lower apparent OM reactivity.

The inversely determined values of the shaping parameter *a* control apparent OM reactivity trends along the S–N transect and range from 10 to 100 years (figure 3*b* and table 3). Like the scaling parameter, the spatial distribution of parameter *a* is neither linked to the positions of the sea–sea ice edge or the PF, nor does it correlate with spatial variability of organic carbon burial rates, which are estimated to be higher on the northern shelf [24], or PP rates, which are higher on the southern shelf [7,8]. The emerging spatial distributions in inversely determined *a*-values instead represent long-term trends in sediment and OM deposition and reflect the spatial heterogeneity of the Barents Sea seafloor. The central station B15 displays the highest *a*-value (*a* = 100 years), and thus the lowest apparent bulk OM reactivity (*k*_SWI_ = *v*/*a* = 1.0 × 10^−3 ^yr^−1^) along the S–N transect. By contrast, inversely determined *a*-values at all other sides are an order of magnitude lower (*a* = 10–20 years; table 3) and reflect a generally higher apparent OM reactivity at the SWI (*k*_SWI_ = *v*/*a* = 4.5 × 10^−3^–2.0 × 10^−2^ yr^−1^), but a faster decrease of apparent OM reactivity with burial depth/time [45].

The Barents Sea shelf is characterized by strong seasonality and the occurrence of summer blooms that enhance the flux of fresh, reactive OM from surface waters to the seafloor. The magnitude of these fluxes is strongly coupled to the position of the PF and the summer ice edge [7–9,12,16,18,52]. However, a large fraction of the OM that settles onto the seafloor is efficiently consumed by benthic fauna prior to burial [52]. The buried material is thus likely more representative of the long-term depositional patterns in the Barents Sea rather than of seasonal variations. Our RTM results confirm this notion. The spatial distribution of apparent OM reactivity does not relate to the PP trends south and north of the PF, but rather appears to be controlled by local variations in OM sources, as well as long-term prevailing oceanographic conditions. Additionally, there is evidence of organic carbon adsorption onto reactive iron mineral phases (OC-Fe = 20.0 ± 7.9%OC) [24] which results in OM physical protection and consequently decrease in apparent OM reactivity. Despite the overall marine and highly reactive character of OM buried in the surface sediments [16,23], there is evidence of terrestrial OM input into the Barents Sea shelf [37,53], which is likely pre-aged and less reactive than fresh marine-derived algal detritus. Sediments off SE Spitsbergen (77–78° N) exhibit elevated proportions of terrestrial OM (greater than 50%) within surface sediments [53]. Additionally, the high contribution of terrestrial OM in this area is further supported by high ^137^Cs inventories, which result from terrestrial supply via coastal erosion, glacial and/or sea ice melting near Svalbard [38]. Such terrestrial contribution from Svalbard likely influences OM deposition at the central station B15, although in relatively lower proportions given the reduced ^137^Cs inventories compared to those of Svalbard surroundings [38]. At the same time, the contribution of fresh, marine-derived OM is less evident at the central station B15 compared to the southernmost and northernmost portions of the transect, where the marine-derived signal is better preserved in the uppermost sediment layers. Therefore, the enhanced contribution of pre-aged, physically protected, terrestrially derived OM explains the lower apparent OM reactivity determined for the central Barents Sea seafloor.

### (b) Organic matter reactivity controls on benthic-pelagic coupling

**Figure 4.**
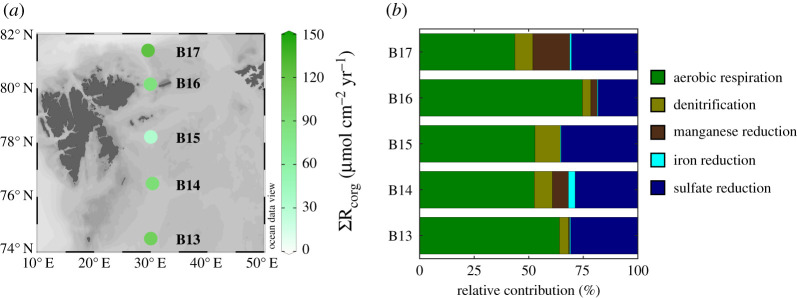
Organic matter degradation dynamics derived from steady-state RTM simulations. (*a*) Depth-integrated rates (upper 100 cm of sediment column) of heterotrophic organic matter degradation; (*b*) relative contribution of heterotrophic metabolic pathway to total organic matter oxidation. See electronic supplementary material, figure S2 for depth evolutions of total rates and relative contributions of each metabolic pathway (electronic supplementary material, table S6). (Online version in colour.)

**Figure 5.**
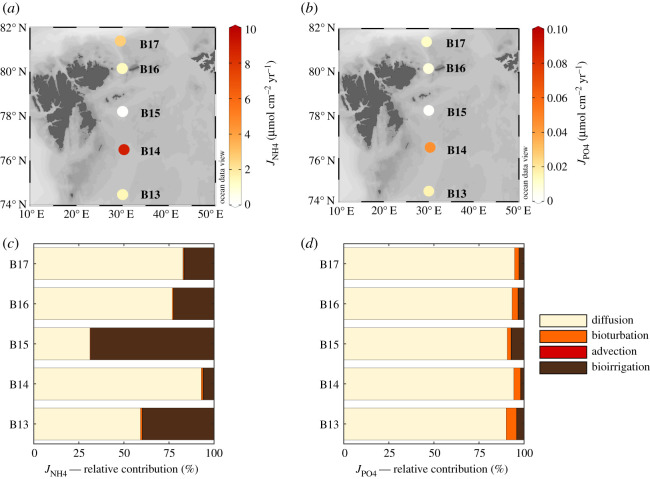
Benthic nutrient fluxes across the sediment-water interface derived from steady-state RTM simulations. (*a*) Total ammonium fluxes, $J_{\text{N}{\text{H}}_{\text{4}}}$; (*b*) total phosphate fluxes, $J_{\text{P}{\text{O}}_{\text{4}}}$; (*c*) relative contributions of transport mechanisms to $J_{\text{N}{\text{H}}_{\text{4}}}$; (*d*) relative contribution of transport mechanisms to $J_{\text{P}{\text{O}}_{\text{4}}}$. See electronic supplementary material, table S7 for relative contributions of each transport mechanism to benthic fluxes. (Online version in colour.)

In addition to inversely determined apparent OM reactivities, the applied RTM approach also allows us to quantify the rates of the coupled diagenetic reaction network that is driven by quantity and quality of OM deposition to the Barents Sea seafloor. Due to the strong coupling between overlapping reactions (see electronic supplementary material, figure S2), these rates are not easily or directly obtained from observations alone and the integrated data-model approach can thus help disentangle and quantify the reaction network. Here, we discuss the rates of OM degradation integrated over the top 100 cm of sediment (figure 4), as well as the ${\text{NH}}_{\text{4}}^{\text{ + }}$ and ${\text{PO}}_{\text{4}}^{\text{3}-}$ benthic fluxes across the SWI (figure 5).

The depth-integrated rates of OM degradation $\left(\sum R_{\text{Corg}}\right)$ range between 31 and 122 µmol C cm^−2^ yr^−1^ (figure 4*a*). Along the S–N transect, $\sum R_{\text{Corg}}$ (figure 4) follows the trend in apparent OM reactivity (the trend in *v* and the inverse trend in *a*; see equation (2.2); table 3), suggesting that reactivity exerts the main control on OM turnover in the sediments. The low rates at central station B15 reflect the burial of less reactive, likely of pre-aged OM at this site. The buried OM has already lost the most reactive fractions prior to deposition and is less available for microbial processing within the sediment. By contrast, the highest $\sum R_{\text{Corg}}$ values are determined for the southernmost station B13, as well as the northernmost station B17 ($\sum R_{\text{Corg}}$ > 100 µmol C cm^−2^ yr^−1^), which are both influenced by AW [5,7–9]. The *v*-values derived for those areas (*v *≥ 0.150) reflect the burial of more reactive OM, with a higher bioavailability [16,52].

Aerobic respiration is the dominant metabolic pathway, contributing to up to 75% of the total respiration in B16. Sulfate reduction is the second most important metabolic pathway, whereas sub-oxic pathways show minor and highly variable contributions to overall OM degradation rates (figure 4*b*; electronic supplementary material, table S6). The contribution of aerobic respiration > 40% is unexpectedly high for shelf sediments that typically range from less than 10% [28] to 17% [48]. Similarly, high aerobic respiration contribution to OM degradation (38%) has also been observed in Greenland coastal sediments associated with intense recycling of OM [54]. Arctic shelf sediments exhibit elevated sediment oxygen demand (10 ± 7.9 mmol O_2 _m^−2^ d^−1^), which is associated with high availability of fresh, high reactive OM [3]. In the Barents Sea, high rates of sediment oxygen demand have been attributed to the deposition of fresh algal detritus (i.e. chlorophyll-*a*) onto the seafloor, which encourages OM processing [51]. Thus, the high contributions of aerobic degradation are the direct result of generally high, yet rapidly decreasing apparent OM reactivities and thus OM reaction rates.

Model results indicate a minor contribution of sub-oxic pathways (5–26%). At first glance, this is at odds with previous incubation experiments of the Barents Sea shelf sediments that have suggested a strong contribution of manganese and iron reduction to the total anaerobic rates of OM degradation (up to 99%) [55,56]. However, the apparent discrepancy between our model results and these incubation results arises from differences between our RTM approach and the experimental set-up developed in previous studies [55,56] that occur due to site-specific conditions and distinct timescales. Our model integrates diagenetic processes within the first 100 cm of the sediment column and thus over timescales of 0–2000 years. In addition, it encompasses a complex reaction network involving Mn and Fe cycling (see electronic supplementary material, tables S1 and S2). The low contribution of Mn and Fe reduction to total OM respiration in our RTM is, depending on the site, controlled by either (i) the low availability of reactive phases in sediments at B13 and B14 or (ii) the strong coupling of reactive phases with the re-oxidation of reduced species at B15–B17 (see below). By contrast, in the laboratory experiments [55,56], sediments were incubated for a short period of time (days), and thus a much shorter timescale. Additionally, incubations were performed in discrete sediment intervals, with no connection between each sediment depth, and only covering shallow sediment depths (less than 15 cm). As such, the experiments do not allow the development of a full redox zonation, and therefore the results are not comparable to our RTM assessments.

Model results also show that, despite a hardly visible depletion of sulfate concentrations with depth (figure 2), sulfate reduction accounts for 18–35% (electronic supplementary material, table S6) of total OM degradation in the upper 100 cm (figure 4*b*), and sulfate reduction rates develop in shallow sediment depth (approx. 3–10 cmbsf; see electronic supplementary material, figure S2). These values compare well with the contribution of sulfate reduction to overall OM degradation in Greenland sediments [54]. OM degradation coupled to sulfate reduction results in the production of sulfides $\text{( H}{\text{S}}^{-}+{\text{H}}_{\text{2}}\text{S})$, which are rapidly re-oxidized by reactive Mn and Fe phases, thus depleting these terminal electron acceptors for OM degradation and/or react with Fe^2+^ to form FeS, and FeS_2_ (for details see reaction network, electronic supplementary material, tables S1 and S2).

Model-derived benthic ammonium $\left(J_{\text{N}{\text{H}}_{4}}\right)$ and phosphate $\left(J_{\text{P}{\text{O}}_{\text{4}}}\right)$ fluxes (table 3) across the SWI are primarily driven by OM reactivity patterns along the S–N transect (figure 5). The central station B15 exhibits the lowest release of those nutrients back to the bottom waters ($J_{\text{N}{\text{H}}_{4}}=0.05\;\mu \text{mol}\;{\text{NH}}_{4}^{+}\;\text{c}{\text{m}}^{-2}\;\text{y}{\text{r}}^{-1}$; $J_{\text{P}{\text{O}}_{4}}=0.001\;\mu \text{mol}\;{\text{PO}}_{4}^{3-}\text{c}{\text{m}}^{-2}\;\text{y}{\text{r}}^{-1}$). Such low fluxes result from both low OM degradation rates (figure 4*a*) and intense recycling of ammonium (nitrification) and phosphate (adsorption and desorption to iron phases) within the sediments. Additionally, RTM simulations show that ammonium adsorption to sediment is high, removing ammonium from porewaters and preventing release across the SWI. Similarly, in the model phosphate experiences strong coupling with reactive iron phases (figure 5*b*). The combination of high availability of reactive Fe phases and high rates of P–Fe adsorption contributes to the low phosphate fluxes in B15. By contrast, B14 displays the highest ammonium and phosphate fluxes $\left(J_{\text{N}{\text{H}}_{4}}=8.85\;\mu \text{mol}\;{\text{ NH}}_{\text{4}}^{\text{ + }}\;\text{c}{\text{m}}^{-2}\;\text{y}{\text{r}}^{-1};\;\;J_{\text{P}{\text{O}}_{4}}=0.045\;\mu \text{mol}\;{\text{PO}}_{4}^{3-}\;\text{c}{\text{m}}^{-2}\;\text{y}{\text{r}}^{-1}\right)$. The combination of degradation of reactive OM (figure 4) with low SWI oxygen levels (table 2) results in low rates of nitrification, allowing an intense upward flux and ammonium release back into bottom waters. Similarly, high phosphate fluxes originate from intense OM recycling associated with low recycling of reactive Fe phases, which allows phosphate accumulation in the porewaters and release across the SWI. In the northernmost part of the transect (B16 and B17), due to higher availability and strong recycling of reactive Fe phases, phosphate fluxes are lower than those observed in southernmost part of the transect (B13), where Fe is less available (table 3). Despite the important role benthic nutrient fluxes play in OM cycling, data are limited across arctic shelves, and particularly scarce for the Barents Sea shelf [3]. Overall, the benthic ammonium fluxes quantified in the S–N transect fall within the range of diffusive fluxes observed in other regions of the Barents Sea and Svalbard shelves (0.00–8.76 µmol ${\text{NH}}_{\text{4}}^{\text{ + }}$ cm^−2 ^yr^−1^) [57,58]. Here, low ammonium fluxes are generally associated with efficient ammonium regeneration in the sediment, as well as a strong coupling between nitrification and denitrification at the oxic-anoxic interface [58], whereas the largest fluxes are associated with biologically active sediments [57] in agreement with our findings. Comparative data for phosphate fluxes in the Barents Sea are currently lacking. A recent study investigating phosphorus sedimentary dynamics in two fjords on the coast of Spitsbergen [59] found that organic phosphorus accounts for 60–97% of annual phosphorus fluxes, and the magnitude of such fluxes are quantitatively related to OM supply. Additionally, the authors argue that the low inorganic phosphorus fluxes and the efficient phosphorus burial are attributed to adsorption into mineral surfaces [59]. Although evidence is limited, it supports the controls of OM reactivity and adsorption/desorption processes on phosphate fluxes revealed by our RTM results.

Across the transect, the relative significance of different transport mechanisms for benthic fluxes of ammonium (figure 5*c*) and phosphate (figure 5*d*) is variable (electronic supplementary material, table S7). In general, bioturbation fluxes are negligible for $J_{\text{N}{\text{H}}_{\text{4}}}$ (less than or equal to 1%) and small for $J_{\text{P}{\text{O}}_{\text{4}}}$ (2–6%) and advective fluxes are negligible at all stations. Molecular diffusion exerts the main control on sediment-water exchange fluxes across most stations, contributing greater than 90% of the total $J_{\text{P}{\text{O}}_{\text{4}}}$. By contrast, $J_{\text{N}{\text{H}}_{\text{4}}}$ diffusive fluxes reveal a large variability (31–93%), which is mainly driven by the spatial heterogeneity in apparent OM reactivity. Bioirrigation represents 68% of total fluxes at central station B15, where total $J_{\text{N}{\text{H}}_{\text{4}}}$ are comparatively low and two orders of magnitude smaller than at other sites (table 3). In our model simulations, intense nitrification consumes ammonium in the upper sediment layer and thus reduces the concentration gradient and as a result diffusive flux. As a consequence, bioirrigation and not diffusion is the main driver of porewaters and bottom water exchange through the SWI at the central station B15.

## 4. Conclusion

Distributions of OM reactivity parameters, degradation rates and nutrient fluxes revealed by our RTM approach along an S–N transect highlight strong benthic-pelagic coupling in the Barents Sea. Both southernmost (B13 and B14) and northernmost (B16 and B17) portions of the transect are characterized by the burial of reactive, marine-derived OM. As such, they display intense OM recycling and a strong release of nutrients back to the water column, which could sustain primary production. By contrast, burial of less reactive, terrestrially derived OM at the central portion of the transect (B15) results in a lower OM turnover, a less efficient benthic recycling of nutrients, and low fluxes across the SWI. Those factors limit nutrient return to the water column, and thus could result in lower productivity. Assuming steady-state conditions, benthic-pelagic coupling in the Barents Sea seems to be rather controlled by the spatial heterogeneity of the seafloor, as well as the prevailing oceanographic conditions, instead of spatio-temporal variations in sea ice and seasonal PP dynamics. More specifically, our findings highlight the impact of long-term OM deposition on benthic-pelagic coupling, as well as the influence of AW on PP over the Barents Sea shelf.

Our steady-state approach offers a baseline mechanistic understanding of processes governing OM and nutrient cycling and allows us to quantify benthic processes. This is crucial for further investigations that aim to explore how the Barents Sea seafloor will respond to climate change-driven perturbations to OM productivity and export. Here, we provide the first estimate of apparent OM reactivity parameters across the Barents Sea S–N transect, which can be readily incorporated into sensitivity studies and upscaled to other arctic regions.

## Supplementary Material

Electronic Supplementary Material for Freitas et al. 'Benthic-pelagic coupling in the Barents Sea: an integrated data-model framework'

## Data accessibility

The model reaction network and reaction rates, as well as the data used to inform the data-model fittings are available in the electronic supplementary material. The model code is available at GitHub repository (https://github.com/ChangingArcticOceanSeafloorModelling/Freitas_etal_PhilTransA_BenthicPelagicCouplingBarentsSea).
