# Supplementary material for: Benthic-pelagic coupling in the Barents Sea: an integrated data-model framework
Source: Philos Trans A Math Phys Eng Sci. 2020 Aug 31;378(2181):20190359. doi: 10.1098/rsta.2019.0359 (PMC7481668; doi:10.1098/rsta.2019.0359)
Supplement: Electronic Supplementary Material for Freitas et al. 'Benthic-pelagic coupling in the Barents Sea: an integrated data-model framework' [file rsta20190359supp1.pdf]

Philosophical Transactions of the Royal Society A

Electronic Supplementary Material

Benthic-pelagic coupling in the Barents Sea: an integrated data-model framework

Felipe S. Freitas[*1,2], Katharine R. Hendry[1], Sian F. Henley[3], Johan C. Faust[4], Allyson C. Tessin[4,5], Mark A. Stevenson[6], Geoffrey D. Abbott[6], Christian März[4], Sandra Arndt[2]

http://dx.doi.org/10.1098/rsta.2019.0359

[1] *School of Earth Sciences, University of Bristol, Wills Memorial Building, Queen's Road, Bristol BS8 1RJ UK*

[2] *BGeosys, Department of Earth and Environmental Sciences, CP 160/02, Université Libre de Bruxelles, 1050 Brussels, Belgium*

[3] *School of GeoSciences, University of Edinburgh, James Hutton Road, Edinburgh EH9 3FE, UK*

[4] *Schoof of Earth & Environment, University of Leeds, Leeds LS2 9JT, UK*

[5]*Department of Geology, Kent State University, Kent, OH, 4424, USA*

[6] *School of Natural and Environmental Sciences, Drummond Building, Newcastle University, Newcastle upon Tyne NE1 7RU, UK*

* *Corresponding author: felipe.salesdefreitas@bristol.ac.uk*

doi:10.1098/rsta.2019.0359

Table S1. Reaction network implemented in the organic matter degradation model for the Barents Sea [1,2].

**Reaction**	**Pathway**
	Primary redox reactions
r_1	*Aerobic respiration*
r_2	*Denitrification*
r_3	*HR Manganese reduction*
r_4	*HR Iron reduction*
r_5	*Organoclastic sulfate reduction*
r_6	*Methanogenesis*
	Secondary redox reactions
r_7	*Nitrification*
r_8	*Manganese reoxidation by oxygen*
r_9	*Iron reoxidation by HR manganese*
r_{10}	*Iron reoxidation by PR manganese*
r_{11}	*Iron reoxidation by oxygen*
r_{12}	*Sulfide oxidation by oxygen*
r_{13}	*Sulfide oxidation by HR manganese*
r_{14}	*Sulfide oxidation by PR manganese*
r_{15}	*Sulfide oxidation by HR iron*
r_{16}	*Sulfide oxidation by MR iron*
r_{17}	*Sulfide oxidation by PR iron*
r_{18}	*Anaerobic oxidation of methane (AOM) coupled to sulfate reduction*
r_{19}	*Methane oxidation by oxygen*
r_{20}	*Iron sulfide oxidation by oxygen*
	Other reactions
r_{21}	*HR Mn ageing*
r_{22}	*HR Fe ageing*

HR (Highly Reactive); MR (Moderately Reactive); (PR) Poorly Reactive

Table S2. Stoichiometry and reaction rates implemented in the organic matter degradation model for the Barents Sea [1,2].

	**Stoichiometry**	**Reaction rate**
	Primary redox reactions	
r_1	$(CH_2O)_x(NH_3)_y(H_3PO_4)_z + (x+2y)O_2 + (y+2z)HCO_3^- \rightarrow (x+y+2z)CO_2 + yNH_4^+ + zHPO_4^{2-} + (x+2y+2z)H_2O$	$r_1 = v \cdot (a+age)^{-1} \cdot CH_2O \cdot f_{O_2}$
r_2	$(CH_2O)_x(NH_3)_y(H_3PO_4)_z + \left(\frac{4x+3y}{5}\right)NO_3^- \rightarrow \left(\frac{2x+4y}{5}\right)N_2 + \left(\frac{x-3y+10z}{5}\right)CO_2 + \left(\frac{4x+3y-10z}{5}\right)HCO_3^- + zHPO_4^{2-} + \left(\frac{3x+6y+10z}{5}\right)H_2O$	$r_2 = v \cdot (a+age)^{-1} \cdot CH_2O \cdot f_{NO_3}$
r_3	$(CH_2O)_x(NH_3)_y(H_3PO_4)_z + 2x\,HR\,MnO_2 + (3x+y-2z)CO_2 + (x+y-2z)H_2O \rightarrow 2xMn^{2+} + (4x+y-2z)HCO_3^- + yNH_4^+ + HPO_4^{2-}$	$r_3 = v \cdot (a+age)^{-1} \cdot CH_2O \cdot f_{HR\,MnO_2}$
r_4	$(CH_2O)_x(NH_3)_y(H_3PO_4)_z + 4x\,HR\,Fe(OH)_3 + (7x+y-2z)CO_2 \rightarrow 4xFe^{2+} + (8x+y-2z)HCO_3^- + yNH_4^+ + zHPO_4^{2-} + (3x-y+2z)H_2O$	$r_4 = v \cdot (a+age)^{-1} \cdot CH_2O \cdot f_{HR\,Fe(OH)_3}$
r_5	$(CH_2O)_x(NH_3)_y(H_3PO_4)_z + \frac{x}{2}SO_4^{2-} + (y-2z)CO_2 + (y-2z)H_2O \rightarrow \frac{x}{2}H_2S + (x+y-2z)HCO_3^- + yNH_4^+ + zHPO_4^{2-}$	$r_5 = v \cdot (a+age)^{-1} \cdot CH_2O \cdot f_{SO_4^{2-}}$
r_6	$(CH_2O)_x(NH_3)_y(H_3PO_4)_z + (y-2z)H_2O \rightarrow \left(\frac{x-2y+4z}{2}\right)CO_2 + (y-2z)HCO_3^- + yNH_4^+ + zHPO_4^{2-} + \frac{x}{2}CH_4$	$r_6 = v \cdot (a+age)^{-1} \cdot CH_2O \cdot f_{CH_4}$
	Secondary redox reactions	
r_7	$NH_4^+ + 2O_2 + 2HCO_3^- \rightarrow NO_3^- + 2CO_2 + 3H_2O$	$r_7 = k_7 \cdot NH_4^+ \cdot O_2$
r_8	$Mn^{2+} + \frac{1}{2}O_2 + 2HCO_3^- \rightarrow HR\,MnO_2 + 2CO_2 + H_2O$	$r_8 = k_8 \cdot Mn^{2+} \cdot O_2$
r_9	$2Fe^{2+} + HR\,MnO_2 + 2HCO_3^- + 2H_2O \rightarrow 2\,HR\,Fe(OH)_3 + Mn^{2+} + 2CO_2$	$r_9 = k_9 \cdot Fe^{2+} \cdot HR\,MnO_2$
r_{10}	$2Fe^{2+} + PR\,MnO_2 + 2HCO_3^- + 2H_2O \rightarrow 2\,HR\,Fe(OH)_3 + Mn^{2+} + 2CO_2$	$r_{10} = k_{10} \cdot Fe^{2+} \cdot PR\,MnO_2$
r_{11}	$Fe^{2+} + \frac{1}{4}O_2 + 2HCO_3^- + \frac{1}{2}H_2O \rightarrow HR\,Fe(OH)_3 + 2CO_2$	$r_{11} = k_{11} \cdot Fe^{2+} \cdot O_2$
r_{12}	$H_2S + 2O_2 + 2HCO_3^- \rightarrow SO_4^{2-} + 2CO_2 + 2H_2O$	$r_{12} = k_{12} \cdot (HS^- + H_2S) \cdot O_2$
r_{13}	$H_2S + HR\,MnO_2 + 2CO_2 \rightarrow Mn^{2+} \rightarrow S^0 + 2HCO_3^-$	$r_{13} = k_{13} \cdot (HS^- + H_2S) \cdot HR\,MnO_2$
r_{14}	$H_2S + PR\,MnO_2 + 2CO_2 \rightarrow Mn^{2+} \rightarrow S^0 + 2HCO_3^-$	$r_{14} = k_{14} \cdot (HS^- + H_2S) \cdot HR\,MnO_2$
r_{15}	$H_2S + 2\,HR\,Fe(OH)_3 + 4CO_2 \rightarrow 2Fe^{2+} + S^0 + 4HCO_3^- + H_2O$	$r_{15} = k_{15} \cdot (HS^- + H_2S) \cdot HR\,Fe(OH)_3$
r_{16}	$H_2S + 2\,MR\,Fe(OH)_3 + 4CO_2 \rightarrow 2Fe^{2+} + S^0 + 4HCO_3^- + H_2O$	$r_{16} = k_{16} \cdot (HS^- + H_2S) \cdot MR\,Fe(OH)_3$
r_{17}	$H_2S + 2\,PR\,Fe(OH)_3 + 4CO_2 \rightarrow 2Fe^{2+} + S^0 + 4HCO_3^- + H_2O$	$r_{17} = k_{17} \cdot (HS^- + H_2S) \cdot PR\,Fe(OH)_3$
r_{18}	$CH_4 + CO_2 + SO_4^{2-} \rightarrow 2HCO_3^- + H_2S$	$r_{18} = k_{18} \cdot CH_4 \cdot SO_4^{2-}$
r_{19}	$CH_4 + 2O_2 \rightarrow CO_2 + 2H_2O$	$r_{19} = k_{19} \cdot CH_4 \cdot O_2$
r_{20}	$FeS + O_2 \rightarrow Fe^{2+}SO_4^{2-}$	$r_{20} = k_{20} \cdot FeS \cdot O_2$
	Other reactions	
r_{21}	$HR\,MnO_2 \rightarrow PR\,MnO_2$	$r_{21} = k_{21} \cdot HR\,MnO_2$
r_{22}	$HR\,Fe(OH)_3 \rightarrow MR\,Fe(OH)_3$	$r_{22} = k_{22} \cdot HR\,Fe(OH)_3$

Table S3. General model parameters implemented in the organic matter degradation model for the Barents Sea.

**Parameter**		**Unit**	**Value**	**Reference**
		Transport parameters		
Length of model domain	L	cm	100	*This study*
Bioirrigation coefficient	a_0	yr^{-1}	10	[2]
Bioirrigation attenuation depth	x_{irri}	cm	3.5	[2]
Oxygen molecular diffusion coefficient	D_{O_2}	$cm^2\ yr^{-1}$	380.44	[3]
Nitrate molecular diffusion coefficient	$D_{NO_3^-}$	$cm^2\ yr^{-1}$	394.58	[3]
Sulfate molecular diffusion coefficient	$D_{SO_4^{2-}}$	$cm^2\ yr^{-1}$	173.92	[3]
Ammonium molecular diffusion coefficient	$D_{NH_4^+}$	$cm^2\ yr^{-1}$	395.87	[3]
Phosphate molecular diffusion coefficient	$D_{PO_4^{3-}}$	$cm^2\ yr^{-1}$	112.35	[3]
Manganese molecular diffusion coefficient	$D_{Mn^{2+}}$	$cm^2\ yr^{-1}$	123.38	[3]
Iron molecular diffusion coefficient	$D_{Fe^{2+}}$	$cm^2\ yr^{-1}$	136.24	[3]
Hydrogen sulfide molecular diffusion coefficient	D_{H_2S}	$cm^2\ yr^{-1}$	331.61	[3]
Porosity	φ	–	*Site-specific*	*See table S4*
Bioturbation coefficient	D_{bio}	$cm^2\ yr^{-1}$	*Site-specific*	*See table S4*
Bioturbation depth	z_{bio}	cm	*Site-specific*	*See table S4*
Sedimentation rate	ω	$cm\ yr^{-1}$	*Site-specific*	*See table S4*
		Reaction parameters		
Stoichiometry constants	$x/y/z$	–	106/12/1	[2]
OM Scaling parameter	v	–	*variable*	[4]
OM Shaping parameter	a	yr	*variable*	[4]
OM reactivity – multi-G	k_{MG}	yr^{-1}	$10^{-15} - -\log(a) + 2$	*This study*
OM age	age	yr	*variable*	[5]
Oxygen half-saturation constant	K_{O_2}	M	$8.0 \cdot 10^{-9}$	[3]
Nitrate half-saturation constant	$K_{NO_3^-}$	M	$5.0 \cdot 10^{-9}$	[3]
Manganese half-saturation constant	$K_{HR\,MnO_2}$	M	$5.0 \cdot 10^{-6}$	[3]
Iron half-saturation constant	$K_{HR\,Fe(OH)_3}$	M	$1.25 \cdot 10^{-5}$	[3]
Sulfate half-saturation constant	$K_{SO_4^{2-}}$	M	$1.0 \cdot 10^{-7}$	[3]

doi:10.1098/rsta.2019.0359

Table S4. Site-specific transport parameters adopted at each studied location along a 30°E S–N transect in the Barents Sea.

**Parameter**		**Unit**	**B13**	**B14**	**B15**	**B16**	**B17**
Porosity at sediment-water interface	φ	–	0.89	0.91	0.92	0.82	0.82
Porosity at depth	φ_∞	–	0.62	0.71	0.62	0.50	0.62
Porosity attenuation	φ_{att}	–	0.15	0.18	0.10	0.10	0.10
Bioturbation coefficient	D_{bio} [6]	$cm^2\ yr^{-1}$	6.0	4.0	2.0	2.5	2.0
Bioturbation depth	z_{bio} [6]	cm	2	4	5	5	4
Sedimentation rate	ω [7]	$cm\ yr^{-1}$	0.05	0.05	0.06	0.05	0.05
Temperature	T [8]	ºC	1.76	1.94	–1.50	–1.45	1.75
Salinity	S [8]	–	35	35	35	35	35
Water depth	h [8]	m	355	290	330	294	291

doi:10.1098/rsta.2019.0359

Table S5. Site-specific reaction parameters determined in this study for each studied location along a 30°E S–N transect in the Barents Sea. Parameters correspond to reaction network outlined in Table S1 and Table S2.

Parameter	Unit	B13	B14	B15	B16	B17
k_7	M^{-1} yr^{-1}	$1.5 \cdot 10^{7}$	$1.5 \cdot 10^{7}$	$1.5 \cdot 10^{11}$	$1.5 \cdot 10^{9}$	$1.5 \cdot 10^{10}$
k_8	M^{-1} yr^{-1}	$2.0 \cdot 10^{8}$	$2.0 \cdot 10^{8}$	$2.0 \cdot 10^{8}$	$2.0 \cdot 10^{9}$	$2.0 \cdot 10^{10}$
k_9	M^{-1} yr^{-1}	$2.0 \cdot 10^{3}$	$2.0 \cdot 10^{3}$	$2.0 \cdot 10^{3}$	$2.0 \cdot 10^{3}$	$2.0 \cdot 10^{3}$
k_{10}	M^{-1} yr^{-1}	$2.0 \cdot 10^{4}$	$2.0 \cdot 10^{4}$	$2.0 \cdot 10^{3}$	$2.0 \cdot 10^{3}$	$2.0 \cdot 10^{3}$
k_{11}	M^{-1} yr^{-1}	$1.0 \cdot 10^{9}$	$1.0 \cdot 10^{9}$	$1.0 \cdot 10^{10}$	$1.0 \cdot 10^{9}$	$1.0 \cdot 10^{9}$
k_{12}	M^{-1} yr^{-1}	$1.0 \cdot 10^{9}$	$1.0 \cdot 10^{9}$	$1.0 \cdot 10^{9}$	$1.0 \cdot 10^{9}$	$1.0 \cdot 10^{9}$
k_{13}	M^{-1} yr^{-1}	$1.0 \cdot 10^{7}$	$1.0 \cdot 10^{7}$	$1.0 \cdot 10^{7}$	$1.0 \cdot 10^{7}$	$1.0 \cdot 10^{7}$
k_{14}	M^{-1} yr^{-1}	$1.0 \cdot 10^{4}$	$1.0 \cdot 10^{4}$	$1.0 \cdot 10^{4}$	$1.0 \cdot 10^{4}$	$1.0 \cdot 10^{4}$
k_{15}	M^{-1} yr^{-1}	$1.0 \cdot 10^{7}$	$1.0 \cdot 10^{7}$	$1.0 \cdot 10^{7}$	$1.0 \cdot 10^{7}$	$1.0 \cdot 10^{7}$
k_{16}	M^{-1} yr^{-1}	$1.0 \cdot 10^{6}$	$1.0 \cdot 10^{6}$	$1.0 \cdot 10^{6}$	$1.0 \cdot 10^{6}$	$1.0 \cdot 10^{6}$
k_{17}	M^{-1} yr^{-1}	$1.0 \cdot 10^{3}$	$1.0 \cdot 10^{3}$	$1.0 \cdot 10^{3}$	$1.0 \cdot 10^{3}$	$1.0 \cdot 10^{3}$
k_{18}	M^{-1} yr^{-1}	$5.0 \cdot 10^{6}$	$5.0 \cdot 10^{6}$	$5.0 \cdot 10^{6}$	$5.0 \cdot 10^{6}$	$5.0 \cdot 10^{6}$
k_{19}	M^{-1} yr^{-1}	$1.0 \cdot 10^{13}$	$1.0 \cdot 10^{13}$	$1.0 \cdot 10^{13}$	$1.0 \cdot 10^{13}$	$1.0 \cdot 10^{13}$
k_{20}	M^{-1} yr^{-1}	$1.0 \cdot 10^{9}$	$1.0 \cdot 10^{9}$	$1.0 \cdot 10^{9}$	$1.0 \cdot 10^{9}$	$1.0 \cdot 10^{9}$
k_{21}	M^{-1} yr^{-1}	$6.0 \cdot 10^{-1}$	$6.0 \cdot 10^{-1}$	$6.0 \cdot 10^{-1}$	$6.0 \cdot 10^{-1}$	$6.0 \cdot 10^{-1}$
k_{22}	M^{-1} yr^{-1}	$6.0 \cdot 10^{-1}$	$6.0 \cdot 10^{-1}$	$6.0 \cdot 10^{-1}$	$6.0 \cdot 10^{-1}$	$6.0 \cdot 10^{-1}$

doi:10.1098/rsta.2019.0359

Table S6. Model-derived relative contributions of each metabolic pathway to total rates of heterotrophic organic matter degradation.

**Site**	**Aerobic respiration** %	**Denitrification** %	**Manganese reduction** %	**Iron reduction** %	**Sulfate reduction** %
B13	64.1	4.2	0.5	0.5	30.8
B14	52.6	8.3	7.3	3.1	28.7
B15	52.9	11.6	0.01	0.4	35.1
B16	74.6	3.8	2.9	0.4	18.2
B17	43.6	8.2	17.0	0.8	30.4

doi:10.1098/rsta.2019.0359

Table S7. Model-derived relative contributions of each transport pathway to total ammonium and phosphate benthic fluxes across the sediment-water interface.

	Ammonium – J_{NH4}				Phosphate – J_{PO4}			
**Site**	**Diffusion %**	**Bioturbation %**	**Bioirrigation %**	**Advection %**	**Diffusion %**	**Bioturbation %**	**Bioirrigation %**	**Advection %**
B13	59.2	1.1	39.7	<0.01	90.1	5.9	4.0	<0.01
B14	92.9	1.1	6.0	<0.01	94.3	3.9	1.8	<0.01
B15	31.2	0.2	68.6	<0.01	90.7	2.2	7.1	<0.01
B16	76.8	0.8	22.4	<0.01	93.4	3.4	3.2	<0.01
B17	82.8	0.6	16.6	<0.01	94.8	2.4	2.9	<0.01

doi:10.1098/rsta.2019.0359

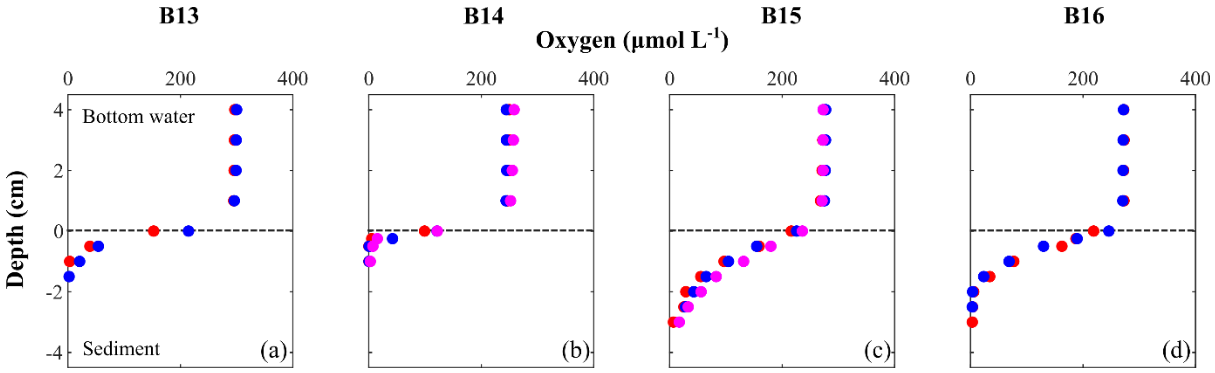

Figure S1. Oxygen concentration profiles measured in bottom waters and sediments along the 30°E S–N transect in the Barents Sea in July 2019 (*RRS* James Clark Ross – JR18006) [9]. Profiles were determined in 2–3 intact cores containing visually undisturbed bottom water and surface sediments from independent megacorer deployments (coloured dots) at each station. Depth profiles measured direct from cores using a *FireSting O2-Mini sensor (Pyro Science)* mounted on a plastic support with a mobile arm which allowed data acquisition at 0.5–1.0 cm scale. Dashed line represents the sediment-water interface. No data available at B17 since this station was inaccessible at the time of sampling.

doi:10.1098/rsta.2019.0359

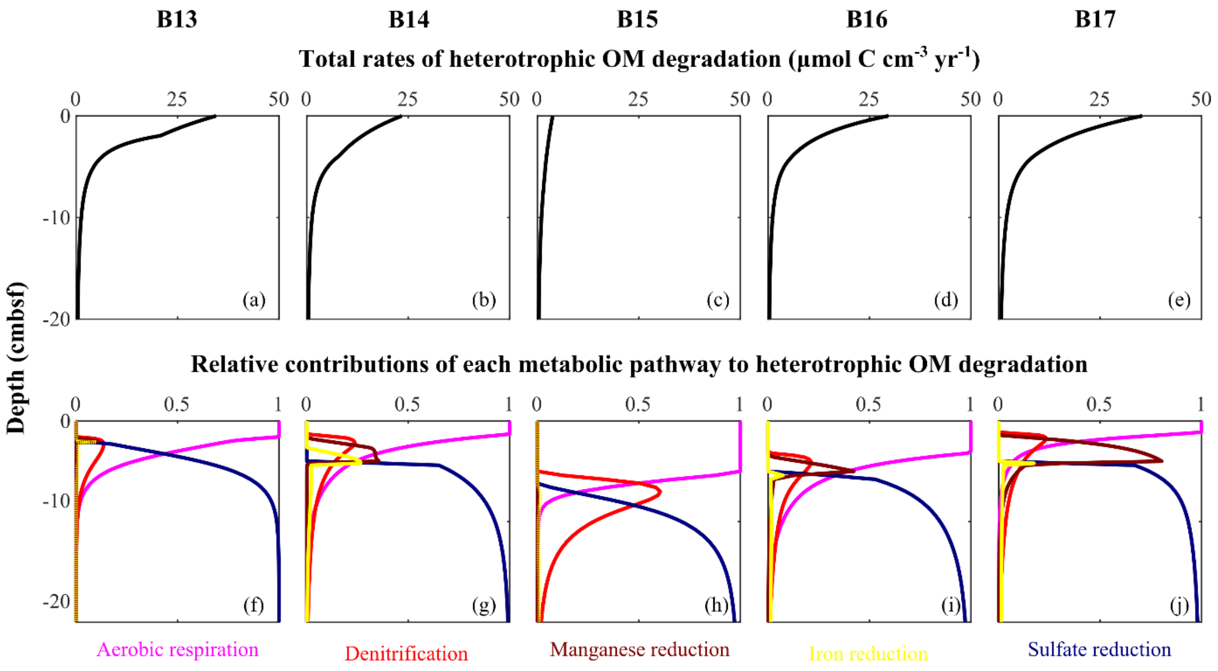

Figure S2. Depth evolution of heterotrophic organic matter degradation rates along the 30°E S–N transect in the Barents Sea. Rates are calculated assuming steady-state conditions and are derived from the primary redox reactions in Table S1 and S2. The top row (a–e) shows the depth profiles of total rates of degradation, and the bottom row (f–j) displays the depth evolution of relative contribution for each respiration pathway.

doi:10.1098/rsta.2019.0359

Table S8. Measured downcore concentration profiles used to inform the data-model fitting at site B13 (cruise JR16006 – July 2017).

			**Porewater**						**Sediment**	
**Event**	**Depth**	NO_3^-	NH_4^+	PO_4^{3-}	Mn^{2+}	Fe^{2+}	**Event**	**Depth**	TOC **(R1)**	TOC **(R2)**
	**cmbsf**	**μM**	**μM**	**μM**	**μM**	**μM**		**cmbsf**	**wt%**	**wt%**
	0.5	12.8	2.1	1.2	0.0	0.0		0.25	2.25	2.201
	1.5	11.9	2.3	1.3	0.0	0.0		0.75	2.061	2.002
	2.5	6.6	9.4	1.2	1.1	9.8		1.25	1.935	1.984
	4.5	0.9	30.1	1.2	1.9	56.2		1.75	1.874	
	6.5	0.6	32.6	1.1	1.3	27.8		2.5	1.829	1.946
	8.5	0.8	39.6	2.0	1.6	26.1		3.5	1.975	1.908
E101	10.5	1.9	24.8	1.2	2.3	26.6		4.5		1.904
	12.5	0.7	30.5	2.6	3.4	52.8		5.5	1.898	1.917
	14.5	0.9	29.7	0.7	3.2	50.6		6.5	1.776	1.796
	16.5	1.0	31.3	0.8	3.0	48.1		7.5	1.802	1.747
	18.5	1.0	36.6	0.5	3.2	54.1	E101	8.5	1.616	1.69
	20.5	0.6	36.7	0.4	4.0	68.7		9.5	1.525	1.432
	25.5	0.5	37.8	0.2	3.8	68.7		10.5	1.399	1.472
								11.5	1.386	1.464
	0.5	9.0	1.1	0.8	0.8	0.0		12.5	1.373	1.458
	1.5	6.4	9.2	1.8	10.9	45.2		13.5	1.361	1.296
	2.5	2.5	27.2	2.6	10.5	83.6		14.5	1.261	1.221
	4.5	1.7	41.3	1.2	8.8	79.1		15.5	1.257	1.237
	6.5	0.9	46.2	1.2	11.3	112.9		16.5	1.042	1.146
	8.5	1.3	40.2	2.2	12.6	113.0		17.5	1.24	1.24
E102	10.5	0.7	39.1	1.8	16.2	102.1		18.5	1.109	1.196
	12.5	2.0	24.7	3.0	7.3	37.9		19.5	1.212	1.208
	14.5	0.6	36.3	2.4	6.1	60.7				
	16.5	0.8	36.5	2.6	5.1	43.8				
	18.5	0.7	41.8	1.5	5.6	55.2				
	20.5	0.5	44.9	0.4	5.3	64.4				
	25.5	0.7	47.5	1.1	4.7	81.3				
	0.5	9.3	1.9	0.8	0.9	0.0				
	1.5	8.6	5.3	1.8	2.7	10.1				
	2.5	4.0	12.7	1.8	3.6	17.4				
	4.5	0.4	40.1	1.0	4.6	67.7				
	6.5	0.7	37.4	2.3	5.2	56.1				
	8.5	0.5	35.0	3.3	6.1	37.7				
E104	10.5	0.6	38.2	4.2	6.8	39.4				
	12.5	0.7	35.8	3.9	8.7	40.6				
	14.5	0.5	37.3	3.9	9.0	35.4				
	16.5	0.5	39.4	4.2	9.7	20.7				
	18.5	0.5	39.7	4.7	6.2	30.7				
	20.5	0.3	40.6	3.4	4.5	33.0				
	25.5	0.7	45.1	4.1	3.5	40.3				
	30.5	0.6	47.0	3.9						

doi:10.1098/rsta.2019.0359

Table S9. Measured downcore concentration profiles used to inform the data-model fitting at site B14 (cruise JR16006 – July 2017).

Porewater

**Event**	**Depth** cmbsf	NO_3^- µM	NH_4^+ µM	PO_4^{3-} µM	Mn^{2+} µM	Fe^{2+} µM
E292	0.5	13.9	3.3	1.5	15.4	
	1.5	4.6	10.3	1.2	16.9	
	2.5	2.4	23.3	2.4	0.0	
	4.5	2.9	22.9	1.8	0.0	
	6.5	1.7	28.2	1.6	1.4	
	8.5	1.6	24.2	1.6	2.8	
	10.5	1.5	22.2	1.2	2.5	
	12.5	1.7	19.4	1.5	2.5	
	14.5	1.5	19.8	1.9	4.6	
	16.5	1.5	19.9	2.4	2.7	
	18.5	1.6	26.2	3.1	2.2	
	20.5	2.0	33.2	3.8	1.8	
	25.5	2.0	44.0	6.4	1.4	
	30.5	2.2	48.7	7.3	1.0	
E294	0.5	12.0	3.3	1.0	3.2	0.0
	1.5	6.1	14.4	1.5	49.1	32.3
	2.5	2.4	32.3	5.2	26.9	126.6
	4.5	1.8	33.8	5.7	15.5	125.2
	6.5	1.4	37.3	6.2	10.1	122.8
	8.5	1.4	38.0	5.6	15.6	111.8
	10.5	1.4	34.1	7.2	6.9	99.6
	12.5	1.6	32.0	4.4	6.2	77.5
	14.5	1.5	40.9	5.2	7.3	105.3
	16.5	1.3	47.8	7.6	7.7	139.1
	18.5	1.5	49.6	6.4	8.8	126.8
	20.5	1.4	56.9	8.1	10.2	128.3
	25.5	1.6	67.9	9.0	10.3	109.9
	30.5	1.7	66.1	9.0	11.1	104.3
E295	0.5	12.8	5.2	1.3	1.9	0.0
	1.5	5.3	19.6	3.2	31.6	91.8
	2.5	2.2	25.6	5.2	24.6	108.1
	4.5	1.6	31.6	5.9	9.6	99.8
	6.5	1.6	26.2	3.4	5.4	62.7
	8.5	1.5	25.4	3.2	3.1	55.9
	10.5	1.7	25.6	2.8	2.8	41.6
	12.5	2.1	24.2	3.4	3.7	34.0
	14.5	1.6	28.4	4.8	2.5	38.2
	16.5	1.6	32.3	4.9	2.4	34.8
	18.5	1.5	37.5	6.2	3.0	41.9
	20.5	1.7	38.2	6.2	2.9	38.7
	25.5	1.5	46.4	7.9	3.5	46.3
	30.5	1.5	50.2	8.4	3.2	45.2

Sediment

**Event**	**Depth** cmbsf	TOC (R1) wt%	TOC (R2) wt%
E295	0.25	2.5	2.5
	0.75	2.3	2.4
	1.25	2.4	2.4
	1.75	2.3	2.4
	2.5	2.4	
	3.5	2.4	2.4
	4.5	2.3	2.2
	5.5	2.4	2.3
	6.5	2.2	2.1
	7.5	2.2	2.2
	8.5	2.2	2.2
	9.5	2.0	2.0
	10.5	2.1	2.0
	11.5	2.1	2.2
	12.5	2.0	2.1
	13.5	2.0	2.0
	14.5	2.0	2.0
	15.5	1.9	
	16.5	2.2	1.9
	17.5	1.9	1.8
	18.5	1.9	1.8
	19.5		1.9
	20.5	1.7	1.8
	21.5	1.7	1.8
	22.5	1.7	1.7
	23.5	1.7	1.8
	24.5	1.6	1.7
	25.5	1.7	1.7
	26.5	1.6	1.6
	27.5	1.6	1.6
	28.5	1.6	1.6
	29.5	1.7	1.7
	30.5	1.7	1.7
	31.5	1.6	1.6
	32.5	1.5	1.5

doi:10.1098/rsta.2019.0359

Table S10. Measured downcore concentration profiles used to inform the data-model fitting at site B15 (cruise JR16006 – July 2017).

Porewater

**Event**	**Depth** cmbsf	NO_3^- µM	NH_4^+ µM	PO_4^{3-} µM	Mn^{2+} µM	Fe^{2+} µM
E144	0.5	12.3	0.6	1.8	0.0	0.0
	1.5	14.0	0.3	1.9	0.0	0.0
	2.5	13.7	0.6	2.2	0.0	0.0
	4.5	7.5	0.2	2.1	5.2	0.0
	6.5	1.3	1.0	2.3	22.4	0.0
	8.5	1.9	3.4	3.1	33.8	0.0
	10.5	1.0	6.9	1.3	43.5	26.5
	12.5	2.1	7.6	0.8	38.8	49.1
	14.5	0.7	10.5	0.7	40.4	84.0
	16.5	1.8	13.5	0.5	45.4	106.1
	18.5	1.2	10.2	0.2	47.1	113.6
	20.5	0.8	22.2	0.8	107.6	102.5
	25.5	0.6	32.4	2.4	99.4	157.2
	30.5	0.5	35.0	1.4	106.0	168.3
E145	0.5	11.0	0.2	1.2	0.0	0.0
	1.5	11.8	0.0	1.3	0.0	0.0
	2.5	11.3	0.1	1.7	0.0	0.0
	4.5	8.5	0.1	2.0	10.4	0.0
	6.5	3.0	1.3	1.8	41.4	0.0
	8.5	1.7	3.8	1.3	55.4	11.9
	10.5	0.6	0.5		69.2	22.1
	12.5	0.5	13.6		59.7	35.9
	14.5	0.9	15.9		66.3	48.8
	16.5	1.0	16.3		78.1	83.0
	18.5	0.5	13.6		90.4	105.0
	20.5					
	25.5					
	30.5					
E146	0.5		1.7	0.3	0.0	0.0
	1.5	11.7	1.1	0.9	0.0	0.0
	2.5	13.5	3.2	1.2	0.0	0.0
	4.5	11.5	3.2	1.4	22.7	0.0
	6.5	4.5	1.5	1.4	104.7	14.9
	8.5	1.7	6.3	1.6	82.2	0.0
	10.5	1.6	9.8	1.7	98.3	0.0
	12.5	1.9	10.0	1.8	93.1	0.0
	14.5	0.9	15.0	1.5	91.3	0.0
	16.5	0.8	17.8	1.1	70.1	192.9
	18.5	0.7	14.8	0.5	74.4	134.6
	20.5	1.2	23.4	0.3	74.3	105.8
	22.5	1.1	26.6	0.1		
	25.5	1.2	19.7	0.2	75.1	78.2
	30.5	1.3	38.9	0.2	75.1	78.2

Sediment

**Event**	**Depth** cmbsf	TOC (R1) wt%	TOC (R2) wt%
E144	0.25	1.7	1.7
	1.25	1.7	1.8
	1.75	1.8	1.9
	2.5	1.8	1.8
	3.5	1.7	1.6
	4.5	1.6	1.6
	5.5	1.5	1.6
	6.5	1.5	1.6
	7.5	1.4	1.4
	8.5	1.4	1.5
	9.5	1.4	1.4
	10.5	1.3	1.3
	11.5	1.4	1.5
	12.5	1.5	1.5
	13.5	1.5	1.5
	14.5	1.5	1.4
	15.5	1.5	1.5
	16.5	1.5	1.5
	17.5	1.5	1.4
	18.5	1.5	1.5
	19.5	1.5	1.5
	20.5	1.4	1.5
	21.5	1.5	1.5
	22.5	1.5	1.4
	23.5	1.5	1.4
	24.5	1.4	1.4
	25.5	1.5	1.5
	26.5	1.4	1.4
	27.5	1.5	1.4
	28.5	1.5	1.5
	29.5	1.4	1.5
	30.5	1.5	1.4
	31.5	1.4	1.5
	32.5	1.3	1.3

doi:10.1098/rsta.2019.0359

Table S11. Measured downcore concentration profiles used to inform the data-model fitting at site B16 (cruise JR16006 – July 2017).

		**Porewater**							**Sediment**	
**Event**	**Depth**	NO_3^-	NH_4^+	PO_4^{3-}	Mn^{2+}	Fe^{2+}	**Event**	**Depth**	TOC **(R1)**	TOC **(R2)**
	**cmbsf**	**µM**	**µM**	**µM**	**µM**	**µM**		**cmbsf**	**wt%**	**wt%**
	0.5	12.1	4.2	1.2	0.0	0.0		0.25		
	1.5	10.5	3.2	1.6	0.0	0.0		0.75	1.6	1.6
	2.5	7.1	5.7	1.3	7.1	0.0		1.25		
	4.5	2.6	15.8	2.2	26.5	77.6		1.75	1.6	1.5
	6.5	1.7	19.8	1.6	31.0	70.2		2.5	1.4	1.4
	8.5	1.3	19.4	1.9	20.5	45.8		3.5	1.4	1.4
	10.5	1.4	20.6	1.7	19.7	51.3		4.5	1.4	1.4
E183	12.5	1.7	21.4	1.2	17.3	54.4		5.5	1.3	1.3
	14.5	1.8	21.8	0.9	20.1	56.7		6.5		
	16.5	2.0	21.6	1.0	23.8	60.4		7.5	1.3	1.3
	18.5	1.7	23.8	0.8	22.3	64.7		8.5	1.2	1.3
	20.5	1.9	26.5	0.9	27.1	67.2		9.5	1.2	1.2
	25.5	1.5	32.9	1.1	25.2	97.6		10.5	1.5	1.5
	30.5	1.4	36.7	2.0	23.0	97.6		11.5	1.1	1.2
							E183	12.5	1.1	1.1
	0.5	11.5	2.6	0.9	0.0	0.0		13.5	1.1	1.2
	1.5	9.4	2.7	1.2	1.2	0.0		14.5	1.1	1.1
	2.5	8.4	4.0	1.4	8.5	0.0		15.5	1.1	1.2
	4.5	4.4	11.2	1.5	27.8	22.0		16.5	1.1	1.1
	6.5	1.4	23.6	1.5	38.4	57.7		17.5	1.1	1.1
	8.5	1.6	26.1	2.0	34.4	69.2		18.5	1.1	1.1
E184	10.5	1.5	31.2	1.7	28.6	81.5		19.5	1.1	1.1
	12.5	1.4	29.5	1.4	27.3	78.3		20.5		1.1
	14.5	2.0	33.7	1.3	28.4	87.9		21.5	1.0	0.9
	16.5	2.1	34.6	1.5	27.8	89.7		22.5	1.1	1.1
	18.5	1.3	37.6	1.4	32.9	115.4		23.5	1.0	1.0
	20.5	1.4	33.9	0.6	23.7	87.9		24.5	1.0	1.0
	25.5	1.4	34.6	0.7	24.2	81.6		25.5	1.0	1.0
								26.5	1.1	1.1
	0.5	12.2	5.2	1.1	0.0	0.0				
	1.5	8.4	3.9	1.0	0.0	0.0				
	2.5	6.4	4.7	1.3	6.2	0.0				
	4.5	1.8	25.9	1.2	17.4	54.1				
	6.5	2.3	14.9	1.1	14.9	28.7				
	8.5	1.4	19.5	0.9	18.8	43.6				
E185	10.5	5.7	24.2	1.2	16.0	61.7				
	12.5	1.7	29.8	1.2	18.5	70.0				
	14.5	1.6	29.5	0.9	20.0	70.8				
	16.5	2.2	30.7	1.2	22.4	71.0				
	18.5	2.0	33.0	1.1	23.2	76.7				
	20.5	1.3	39.0	0.8	26.0	108.1				
	25.5	1.3	37.9	1.3	27.8	111.6				

doi:10.1098/rsta.2019.0359

Table S12. Measured downcore concentration profiles used to inform the data-model fitting at site B17 (cruise JR16006 – July 2017).

	Porewater						Sediment			
**Event**	**Depth cmbsf**	NO_3^- **μM**	NH_4^+ **μM**	PO_4^{3-} **μM**	Mn^{2+} **μM**	Fe^{2+} **μM**	**Event**	**Depth cmbsf**	TOC **(R1) wt%**	TOC **(R2) wt%**
E223	0.5	12.1	0.4	0.8	0.0	0.0	E226	0.3	1.6	1.7
	1.5	12.5	0.4	2.0	0.0	0.0		0.5	1.7	1.8
	2.5	3.3	3.9	1.9	65.4	0.0		1.3	1.6	1.7
	4.5	1.4	16.4	3.5	108.1	110.4		1.8	1.7	1.7
	6.5	0.8	23.7	1.3	118.2	182.0		2.5	1.5	1.6
	8.5	1.2	25.3	4.3	122.1	117.0		3.5	1.4	1.5
	10.5	1.2	34.9	3.8	110.7	133.5		4.5	1.4	1.4
	12.5	1.3	33.4	3.3	97.9	196.1		5.5	1.2	1.3
	14.5	0.8	32.5	1.7	77.3	152.8		6.5	1.1	1.2
	16.5	0.9	35.0	7.8	80.5	169.4		7.5	1.1	1.2
	18.5	0.8	34.8	2.1	75.6	150.8		8.5	1.1	1.1
	20.5	1.1	33.3	2.4	72.0	126.0		9.5	1.0	1.1
	25.5	1.8	35.6	1.5	68.9	99.6		10.5	1.1	1.0
	30.5	0.3	14.0	0.3	53.4	125.3		11.5	1.0	1.0
								12.5	1.0	0.9
E225	0.5	11.6	1.4	1.0	0.0	0.0		13.5	1.0	1.0
	1.5	9.8	0.5	1.7	0.0	0.0		14.5	1.1	1.0
	2.5	6.3	2.8	1.6	35.9	0.0		15.5	1.1	1.2
	4.5	0.8	21.5	2.8	102.2	184.1		16.5	1.1	
	6.5	0.7	29.4	6.2	107.0	189.4		17.5	1.0	1.1
	8.5	1.0	30.1	3.1	99.8	191.6		18.5	1.0	1.1
	10.5	0.7	35.1	5.6	87.2	211.9		19.5	1.0	1.0
	12.5	1.2	40.4	2.2	79.8	212.6		20.5	0.9	0.9
	14.5	0.9	41.5	3.7	80.4	200.5		21.5	0.8	0.9
	16.5	1.5	42.7	2.7	75.1	192.8		22.5	0.9	0.9
	18.5	0.9	47.0	3.6	69.7	185.8		23.5	0.9	0.9
	20.5	0.9	59.8	4.1	66.1	183.6		24.5	0.9	0.9
	25.5	0.8	51.2	4.5	63.4	172.2		25.5	0.9	0.9
	30.5	1.0	50.9	3.8	62.4	158.8		26.5	0.9	0.9
								27.5	0.9	0.9
E226	0.5	11.3	0.0	0.6	0.0	0.0		28.5	0.8	0.8
	1.5	14.0	2.3	1.2	3.0	0.0		29.5	0.8	0.8
	2.5	4.7	11.8	1.3	54.8	0.0		30.5	0.8	0.8
	4.5	1.1	18.4	2.5	180.0	57.5		31.5	0.7	0.7
	6.5	0.9	32.1	5.6	151.4	187.5		32.5	0.7	0.8
	8.5	1.0	34.5	5.0	137.3	198.6		33.5	0.8	0.8
	10.5	1.2	35.4	5.9	120.9	164.3				
	12.5	1.3	43.0	2.3	98.8	224.1				
	14.5	1.0	45.3	3.6	96.2	208.5				
	16.5	0.8	39.7	1.9	96.4	211.7				
	18.5	1.0	49.0	4.3	91.4	198.8				
	20.5	0.8	48.6	2.7	83.5	207.1				
	25.5	1.0	46.6	2.7	75.6	175.0				
	30.5	1.0	59.6	2.7	68.6	152.3				

References

1. Aguilera DR, Jourabchi P, Spiteri C, Regnier P. 2005 A knowledge-based reactive transport approach for the simulation of biogeochemical dynamics in Earth systems. *Geochem. Geophys. Geosystems* **6**, 1–18. (doi:10.1029/2004GC000899)

2. Thullner M, Dale AW, Regnier P. 2009 Global-scale quantification of mineralization pathways in marine sediments: A reaction-transport modeling approach. *Geochem. Geophys. Geosystems* **10**, 1–24. (doi:10.1029/2009GC002484)

3. Van Cappellen P, Wang Y. 1996 Cycling of iron and manganese in surface sediments; a general theory for the coupled transport and reaction of carbon, oxygen, nitrogen, sulfur, iron, and manganese. *Am. J. Sci.* **296**, 197–243. (doi:10.2475/ajs.296.3.197)

4. Boudreau BP, Ruddick BR. 1991 On a reactive continuum representation of organic matter diagenesis. *Am. J. Sci.* **291**, 507–538. (doi:10.2475/ajs.291.5.507)

5. Mogollón JM, Dale AW, Fossing H, Regnier P. 2012 Timescales for the development of methanogenesis and free gas layers in recently-deposited sediments of Arkona Basin (Baltic Sea). *Biogeosciences* **9**, 1915–1933. (doi:10.5194/bg-9-1915-2012)

6. Solan M, Ward ER, Wood CL, Reed AJ, Grange LJ, Godbold JA. In press. Benthic biodiversity-function relations transition across the Barents Sea Polar Front. *Phil. Trans. R. Soc. A.*

7. Zaborska A, Carroll J, Papucci C, Torricelli L, Carroll ML, Walkusz-Miotk J, Pempkowiak J. 2008 Recent sediment accumulation rates for the Western margin of the Barents Sea. *Deep Sea Res. Part II Top. Stud. Oceanogr.* **55**, 2352–2360. (doi:10.1016/j.dsr2.2008.05.026)

8. Dumont E, Brand T, Hopkins J. 2019 CTD data from NERC Changing Arctic Ocean Cruise JR16006 on the RRS James Clark Ross, Jun-August 2017. (doi:doi:10.5285/89a3a6b8-7223-0b9c-e053-6c86abc0f15d)

9. Barnes DKA. 2019 Changing Arctic Ocean Seafloor JR18006 Cruise Report. , 106.
